# The reason why orthopaedic surgeons perform total knee replacement: results of a randomised study using case vignettes

**DOI:** 10.1007/s00167-015-3961-5

**Published:** 2016-01-12

**Authors:** W. C. Verra, K. Q. Witteveen, A. B. Maier, M. G. J. Gademan, H. M. J. van der Linden, R. G. H. H. Nelissen

**Affiliations:** 1 Department of Orthopaedic Surgery, Leiden University Medical Center, PO Box 9600, 2300 RC Leiden, The Netherlands; 2 Section of Gerontology and Geriatrics, Department of Internal Medicine, VU University Medical Center, Amsterdam, The Netherlands; 3 Department of Clinical Epidemiology, Leiden University Medical Center, Leiden, The Netherlands

**Keywords:** Total knee replacement, Indication, Age, Pain, Osteoarthritis

## Abstract

**Purpose:**

End-stage knee osteoarthritis (OA) results in total knee arthroplasty (TKA) surgery. The decision to perform TKA is not well defined, resulting in variation of indications among orthopaedic surgeons. Non-operative treatment measures are often not extensively used. Aim of this study was to investigate factors influencing the decision to perform TKA by Dutch orthopaedic surgeons.

**Methods:**

Three case vignettes, each case divided into two versions, being identical except for information on age (younger and older age), pain (mild and severe pain) or radiological OA (low and high grade) were developed. A questionnaire including these three case vignettes was sent to 599 Dutch orthopaedic surgeons, who were randomised to either one of the two versions. The orthopaedic surgeons were asked whether TKA would be the next step in treatment. Furthermore, from a list of patient factors they were asked how strong these factors would influence the decision to perform TKA.

**Results:**

54 % of the orthopaedic surgeons completed the questionnaire (*n* = 326). Orthopaedic surgeons indicated to perform TKA significantly more often at higher age (73.3 vs. 45.5 %, *p* < 0.001). In the presence of mild pain, orthopaedic surgeons were slightly more reluctant to perform a TKA compared to severe pain (57.0 vs. 64.0 %, n.s.). Mild radiological OA made surgeons more reluctant to perform TKA compared to severe OA (9.7 vs. 96.9 %, *p* < 0.001).

**Conclusion:**

Old age and severe radiological OA are variables which are considered to be important in the decision to perform a TKA. Pain symptoms of moderate or severe pain are unequivocal when considering a TKA.

**Level of evidence:**

Economic/decision analysis, Level III.

**Electronic supplementary material:**

The online version of this article (doi:10.1007/s00167-015-3961-5) contains supplementary material, which is available to authorized users.

## Introduction

Knee osteoarthritis (OA) is a major cause of disability and functional limitations which affects millions of people in our ageing population worldwide [2, 21]. A total knee arthroplasty (TKA) is generally accepted to be an effective surgical treatment for end-stage knee OA [3, 7, 27]. Until now, no succinct criteria on decision-making on TKA are available, other than ‘enough pain’ [7]. The latter not only results in variation among orthopaedic surgeons in their decision to perform a TKA, but also in a potentially large percentage of patients not receiving adequate conservative (i.e. non-operative) treatment for knee OA [1, 16, 20, 25]. On the other hand, not all patients improve after TKA; a study from the Swedish arthroplasty register showed that 17–25 % of the patients after primary TKA were not satisfied or were uncertain about the functionality of their TKA [26]. Since patient expectations on their TKA surgery are not entirely met, well-timed surgery and pre-operative counselling seem to be important variables to be addressed, even more considering the high prevalence of TKA surgery, with about 22,000 cases in 2012 in a small country such as the Netherlands and 719,000 cases in the USA in 2010 [17, 22].

Pain and the degree of radiographic OA are considered important variables in the decision process to perform knee replacement surgery [7, 9, 16, 19]. Pre-operative pain is a strong predictor of postoperative outcome; patients with severe pre-operative pain complaints had worse postoperative outcomes compared to those with less severe pain complaints [5, 10]. On the contrary, patients with mild radiological OA showed little improvement in clinical symptoms compared to patients with severe radiological OA [13]. Most orthopaedic surgeons consider a TKA in case of moderate to severe radiological OA, but there is a well-known weak association between pain symptoms/functional impairment and radiological OA [19, 28]. As for total hip arthroplasty (THA), ranking determinants for their importance in the decision to perform surgery showed that radiological changes were of less importance than pain at rest and at night and/or pain during activities, functional impairment or a decreased range of motion (ROM) [4].

This emphasises the need to explore the variables being involved in the decision-making process to perform TKA. The aim of this study was to evaluate how these factors influence the opinion of Dutch orthopaedic surgeons in the decision to recommend TKA surgery in a given patient. We have used case vignettes to mimic clinical practice; this has never been done before. We hypothesised that Dutch orthopaedic surgeons would recommend TKA to patient with high-grade radiological OA, high levels of pain and older age.

## Materials and methods

In April 2012, all 599 actively practicing orthopaedic surgeons in the Netherlands who were member of the Dutch Orthopaedic Association (NOV) were contacted by e-mail from the NOV to participate. After 2 and 4 weeks, a reminder was sent by e-mail to those who did not respond. All orthopaedic surgeons were randomised into two groups, both groups filled out a different version of a case vignette (version A or B, see below). Randomisation lists were generated randomly by a computer.

### Questionnaire

The web-based survey used in this study was partially based on questionnaires previously used in surveys among orthopaedic surgeons studying different outcomes [4, 18, 19]. In addition, one part of the questionnaire was adapted from a study on geriatric oncology patients [6]. This study used case vignettes with different versions to explore the influence of older age on oncologists’ cancer management [6]. The TKA indication questionnaire was designed and critically appraised by two experienced knee specialists (RN and EL). Before the final versions were distributed to the Dutch orthopaedic surgeons, a pilot test was performed among a test panel of twelve orthopaedic surgeons and residents for final feedback. The software used to distribute the questionnaire was NetQ (NetQuestionnaires Nederland BV, Amsterdam, The Netherlands).

 The questionnaire was divided into three parts: part one consisted of general information of the respondent (gender, employment location (university medical centre, general hospital (private group or fixed salary) or specialised private clinic), number of knee replacements performed each year (<50, 50–100 or >100) and years of experience).

Part two consisted of either version A or B of three case vignettes (Appendix A of ESM). The case vignettes of version A and B were entirely identical except for information on (1) age (old vs. young age), (2) severity of pain (mild vs. severe) and (3) radiological OA (mild vs. severe radiological destruction). Case 1 version A described a 54-year-old patient versus version B an 86-year-old patient. Case 2 version A described a patient with mild pain symptoms and version B a patient with severe pain symptoms. Case 3 version A showed a radiograph with mild radiological OA and version B showed a radiograph with severe radiological OA. A radiograph of the knee was present in all three case vignettes. The diagnosis in all cases was primary OA with no other abnormalities in other joints of the lower extremities. Orthopaedic surgeons were asked for each case: Is a total TKA the next step in your treatment? ‘yes or no’. A short explanation in writing of the chosen answer was mandatory.

Part three of the questionnaire contained factors which might affect the decision to perform TKA surgery. These fourteen decision-modifying factors were extracted from the current orthopaedic literature including; high co-morbidity, severe osteoporosis, obesity, dementia, low quality of life due to knee problems, old age, young age, ineffective conservative treatment, limited walking distance, dependent on activities of daily living (ADL) due to knee problems, moderate motivation of the patient, severe pain, severe radiological OA and mild radiological changes [4, 19]. For this part of the questionnaire, the respondents were instructed to select an answer on a five-point Likert scale: strongly against surgery, against surgery, neutral, in favour of surgery and strongly in favour of surgery. The factors explored in the case vignettes of part two were also included in this part to evaluate their importance in relation to other modifying factors. During the questionnaire, it was not possible to return to the previous question.

Since no study patients were involved, official approval of an ethics board was not necessary.

### Statistical analysis

For analysis of the case vignettes, a Chi-squared test was used. The decision-modifying factors of part three of the questionnaire were presented in a 5-point Likert scale. These factors were ranked in hierarchical order of most likely influencing the decision to perform TKA to most unlikely to perform TKA. ‘Strongly in favour of surgery’ and in favour of surgery together as well as ‘strongly against surgery’ and ‘against surgery’ were combined. We performed no sample size calculation since our sample size consisted of a fixed cohort (i.e. all actively practicing orthopaedic surgeons member of the NOV).

All analyses were performed using SPSS for Windows, version 20. Tests were two-tailed, and *p* values <0.05 were considered to be significant.

## Results

### Characteristics of the respondents

**Fig. 1:**
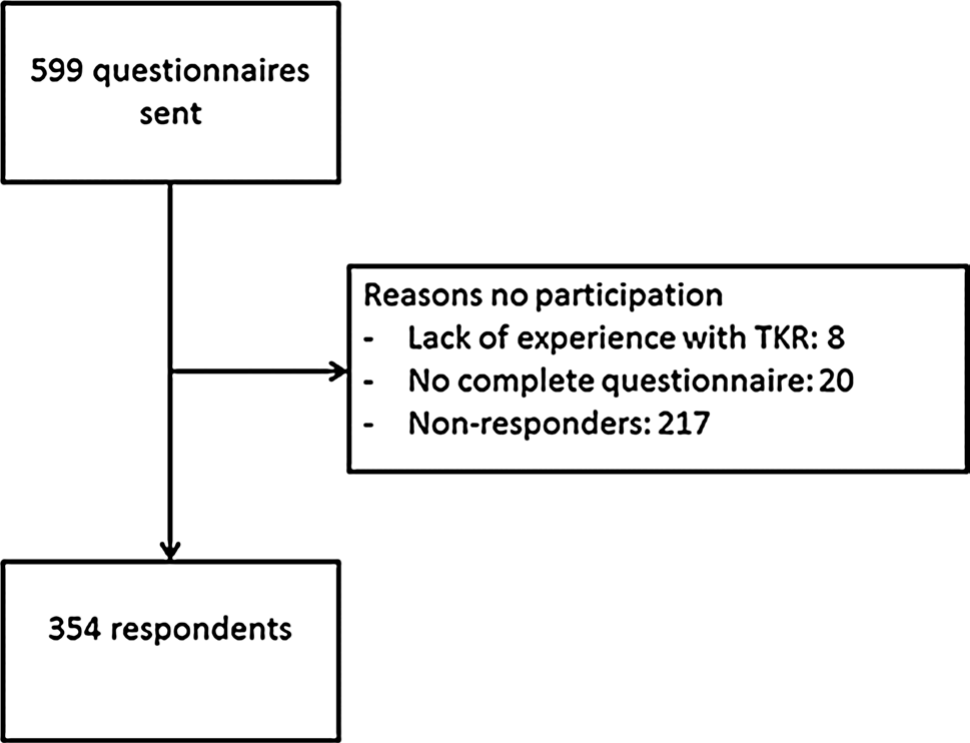
Flow chart participating orthopaedic surgeons

Of the 599 questionnaires, a total of 354 (59 %) orthopaedic surgeons responded after three mailings (Fig. 1). Of the 354 responders, 8 indicated not to participate in the questionnaire due to lack of experience in performing a TKA and 20 did not complete the whole questionnaire. Therefore, 326 (54 %) were included in the analysis. Groups A (*n* = 165) and B (*n* = 161) had comparable general characteristics (Table 1).

**Table 1:** General characteristics of the respondents, stratified by group (*n* = 326)

Characteristics	Group		Total (*n* = 326)
	A (*n* = 165)	B (*n* = 161)	
Gender			
Male (%)	152 (92)	150 (93)	302 (93)
Working environment (%)			
University medical centre	17 (10)	18 (11)	35 (11)
Private practice in general hospital	122 (74)	114 (71)	236 (72)
General hospital (fixed salary)	17 (10)	19 (12)	36 (11)
Specialised knee clinic	9 (6)	10 (6)	19 (6)
Number of knee arthroplasties each year (%)			
<50	50 (30)	59 (37)	109 (34)
50–100	91 (55)	86 (53)	177 (54)
>100	24 (15)	16 (10)	40 (12)
Years of experience (median; IQR)	10 (5–19)	11 (4–20)	10 (5–10)

Values are displayed in frequency (*n*) and percentage (%) if not otherwise indicated

*IQR* interquartile range

### Case vignettes

Case 1, with difference in chronological age, showed that orthopaedic surgeons were willing to perform a TKA more often at higher chronological age (73 vs. 46 %, *p* < 0.0001). Case 2, with difference in severity of pain symptoms, showed no difference in the decision to perform a TKA between the cases with mild and severe pain (57 vs. 64 %, n.s.). Case 3, with difference in radiological knee OA, showed that orthopaedic surgeons were less likely to perform surgery in a patient with mild compared to severe radiological OA (9.7 vs. 96.9 %, *p* < 0.0001) (Table 2).

**Table 2:** Differences in recommendation of TKA, stratified by group dependent on case vignettes

Group		*p* value
A (*n* = 165)	B (*n* = 161)	
Case ‘Age’ 54-year-old patient (45.5 %)	86-year-old patient (73.3 %)	<0.001
Case ‘Pain’ mild pain symptoms (57 %)	Severe pain symptoms (64 %)	n.s.
Case ‘ROA’ mild radiological OA (9.7 %)	Severe radiological OA (96.9 %)	<0.001

(%) The percentages of orthopaedic surgeons who do recommend a TKA in the case vignette. For statistical analysis between the case vignettes, we used a Chi-squared test. Case 1 described a young patient (group A) and old patient (group B). Case 2 described a patient with mild pain symptoms (group A) and severe pain symptoms (group B). Case 3 described a patient with mild radiological OA (group A) and severe radiological OA (group B)

*ROA* radiological osteoarthritis, *n.s.* not significant

If a TKA was not recommended, valgus bracing of the knee, physiotherapy and unicompartmental knee arthroplasties were frequently answered alternatives, but heterogeneity between each of the three case vignettes and the two versions of the questionnaires was seen (Tables 3, 4, 5).

**Table 3:** Explanation not recommending a TKA, case ‘Age’

Case ‘Age’	Group	
	A (*n* = 90)	B (*n* = 43)
High tibial osteotomy	37	–
Unicompartmental knee prosthesis	29	4
Valgus bracing of the knee	17	13
Intra-articular injection	7	11
Expand conservative treatment	6	7
Knee arthroscopy	6	1
Radiographs (long leg)	6	–
MRI	6	–
Physiotherapy	6	5
Patient too young	5	–
Patient too old	–	3
Lateral heel lift	2	–
Lack of information	1	1
Optimise the level of painkillers	1	1

Notes are given in multiple responses (*n*)

**Table 4:** Explanation not recommending a TKA, case ‘Pain’

Case ‘Pain’	Group	
	A (*n* = 71)	B (*n* = 58)
Unicompartmental knee prosthesis	19	22
Valgus bracing of the knee	15	13
Physiotherapy	14	6
Intra-articular injection	11	3
Knee arthroscopy	3	11
High tibial osteotomy	3	7
Lack of information	1	7
No indication for TKR surgery	7	1
Expand conservative treatment	4	1
Optimise the level of painkillers	2	3
Radiographs (stress view)	–	4
MRI	2	1
Watchful waiting	3	–
Lateral heel lift	1	1
Meniscectomy	1	–

Notes are given in multiple responses (*n*)

**Table 5:** Explanation not recommending a TKA, case ‘Radiological OA’

Case ‘Radiological OA’	G	
	A (*n* = 149)	B (*n* = 5)
Discrepancy: complaints versus ROA	47	Not applicable^a^
Intra-articular injection	32	
MRI	24	
Knee arthroscopy	19	
Additional diagnostic testing	17	
Expand conservative treatment	17	
Physiotherapy	12	
Valgus bracing of the knee	7	
Bone scintigraphy	5	
Lack of information	5	
Radiographs (stress view)	4	
Optimise the level of painkillers	4	
X-ray (long leg)	3	
Unicompartmental knee prosthesis	2	
High tibial osteotomy	1	
Expectations too high	1	
Rheumatoid arthritis screening	1	
Weight loss	1	

Notes are given in multiple responses (*n*)

^a^Only 5 respondents who did not recommend a TKR (3.6 % of total)

### Decision-modifying factors

**Fig. 2:**
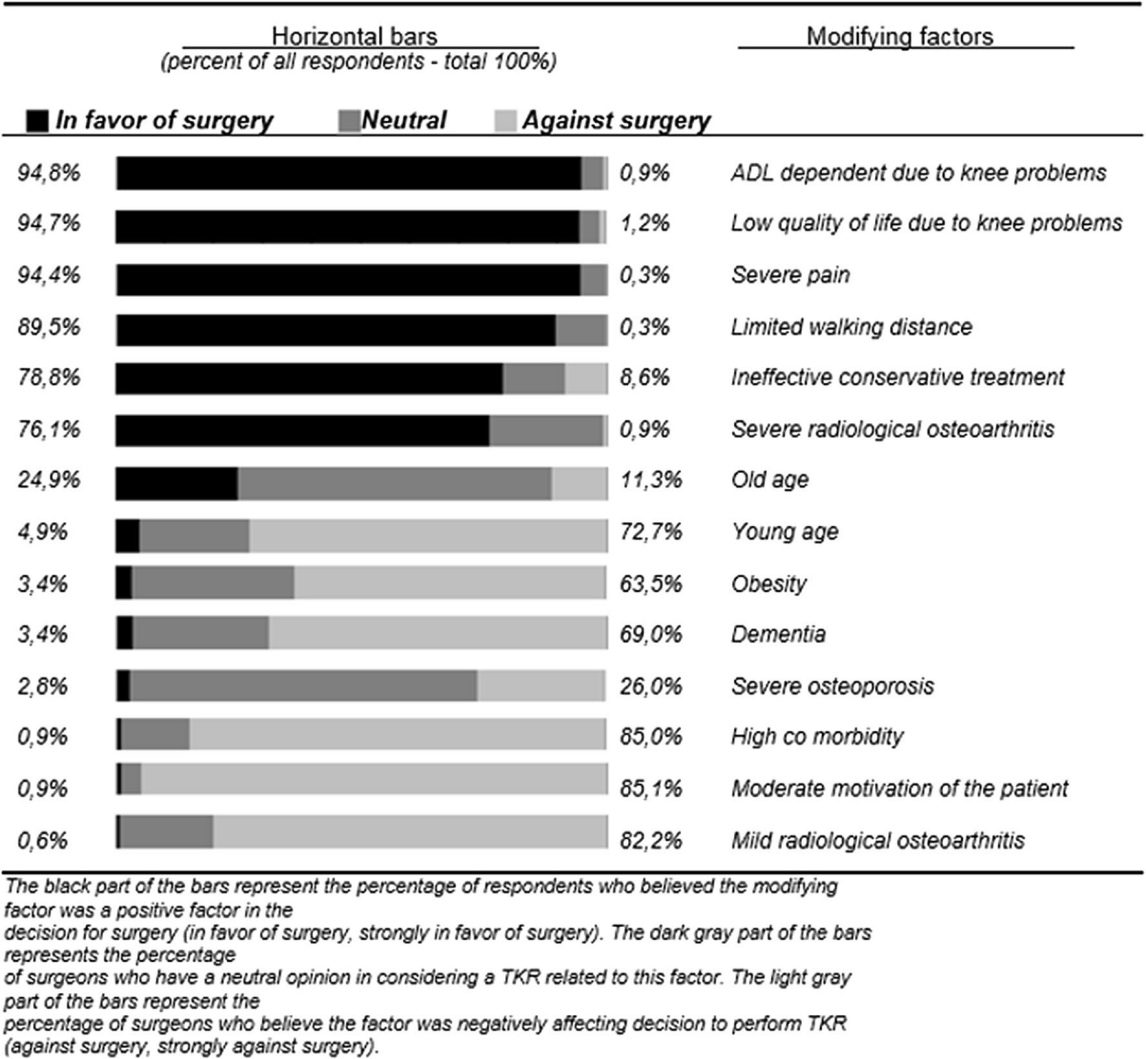
Modifying factors affecting the decision to perform TKA (*n* = 326)

The fourteen patients’ characteristics and modifying factors were ranked in hierarchical order of most likely influencing the decision to perform TKA (Fig. 2). The factors, activities of daily life (ADL) dependency, low quality of life, presence of severe pain, limited walking distance, ineffective conservative treatment and severe radiological OA were positively associated with the decision of orthopaedic surgeons to perform a TKA. On the other hand, mild radiological OA, moderate motivation of the patient, high co-morbidity, dementia and young age urged the orthopaedic surgeons less likely to perform a TKA. Presence of obesity was negatively associated with the decision of the orthopaedic surgeons to perform a TKA, although one-third of the respondents had a neutral opinion about obese patients considering a TKA. Old age and severe osteoporosis were of no clear influence in the decision to perform a TKA.

## Discussion

The most important finding of the present study was older age, and moderate to severe radiological OA was important variable in the decision-making process for TKA by Dutch orthopaedic surgeons, while the level of pain was not strongly associated with the indication to perform a TKA. However, latter is generally considered an important factor to perform TKA. Furthermore, we found that the factors depending on ADL, low quality of life, severe pain, limited walking distance, ineffective conservative treatment and severe radiological OA were associated with the decision of orthopaedic surgeons to perform a TKA.

### Age

Respondents did not consider old age as a contraindication to perform a TKA, but high co-morbidity negatively influenced the decision to perform TKA. Therefore, we assume that relatively good health status is essential for the decision to perform a TKA in aged patients, which is line with the literature [18]. The majority of orthopaedic surgeons delayed recommendation of a TKA in the younger age groups (<55 year), probably due to a higher revision rate within this group and the unpredictable outcome after revision TKA [11, 14]. More than 50 % of the respondents recommended other treatment options for this age group like high tibial osteotomy or unicompartmental knee prostheses, which were suggested most frequently [8, 12, 18, 30].

### Pain symptoms

The current literature highlights the importance of evaluating the pain level experienced by patients in the pre-operative period since less severe pain experienced by patients (i.e. non-catastrophising pain) predicts better postoperative outcome [5, 9, 10]. Differences in pain symptoms (pain at rest, pain at night and pain at activity) did not affect the decision to recommend a TKA in the case vignettes. Based on these results, we can conclude that OA patients presenting with knee pain in the Netherlands are being treated similarly, independent of pain characteristics. However, severe pain is identified by 95 % of the orthopaedic surgeons as a very important variable in the decision to perform a TKA (part three of our study). The OA Research Society International and Outcome Measures in Rheumatology (OARSI-OMERACT) working group has shown that pain and function are weakly predictive in the surgeon’s recommendation for TKA, which underlines our results [7]. Both results are conflicting with the importance of level of knee pain and function pre-operative which strongly affect the postoperative outcome of the patient (less severe knee OA obtain better outcome) [5, 9, 10].

### Radiological OA

Our study showed that the degree of radiological knee OA is an important variable which influences the orthopaedic surgeons’ decision to perform TKA, as was found by others as well [18]. Although ample evidence exists on the discrepancy between the presence of radiological OA and clinical symptoms, most orthopaedic surgeons consider a TKA surgery in the presence of moderate to severe radiological OA [19, 27, 29].

The prevalence of knee OA is increasing, caused by both increasing life span, but also a growing group of people suffering from overweight and therewith negative metabolic changes on the cartilage as well as mechanical overuse of the knee joint [24]. This results in an increase in TKA surgery worldwide, with a predicted increase of over 700 % until 2030 in the USA [15].

Not all patients with a TKA are satisfied. At 1- to 5-year follow-up, about one-fifth of patients with a TKA are not satisfied with their functional outcome [23, 26]. This stresses the importance of pre-operative prediction models on which patients will benefit from a TKA, in order not only to increase quality of life of patients but also to reduce national healthcare costs. With the implementation of patient-reported outcome measures (PROMs) in national registries and the presence of option grids for patients based on prediction models for outcome, the indication for surgery, and thus the variation among orthopaedic surgeons for TKA, is likely to decrease.

Strengths of this study were the relatively large number of respondents, which gives a good reflection of the opinion of the Dutch orthopaedic surgeon. Second, case vignettes with each case developed in two versions were never used before in orthopaedic questionnaires and are an effective method to analyse the current symptoms (age, pain symptoms and radiological OA) determining the decision of an orthopaedic surgeon to perform TKA. With the use of case vignettes, a clinical setting was mimicked, but this virtual setting might still be different from what orthopaedic surgeons actually do in their own clinical practice. Moreover, case vignettes do not give all clinical information, which could affect the decision-making process. For that matter, the influence of conjoined factors in the decision-making process like young age and severe radiological OA and severe pain combined could not be determined. Another limitation was that an inability in the questionnaire existed to select ‘no or less experiences with TKA surgery’, which allows orthopaedic surgeons to finish the questionnaire without noticing they had no or less experiences in knee surgery. However, the latter is also a strong feature if it was a barrier for some respondents to start or complete the questionnaire. Finally, our results are limited to a healthcare system comparable to the Dutch system where surgeons do not receive fee-for-surgery payments or bonus plans (i.e. as an addition to fixed salary employment). These latter factors could also be of important influence in the decision to perform TKA surgery and were not investigated within this study.

Further clinical research is required to clarify the indication criteria of an orthopaedic surgeon for TKA surgery, and prediction models of both the symptom state of patients in the presence of a certain functional deficit and radiological osteoarthritis and the education level of the orthopaedic surgeon will be important variables in such a model. International implementation of the case vignette questionnaire would make cross-cultural differences in indication for TKA among surgeons visible and might define option grids among the different patient groups even better.

## Conclusion

Older chronological aged patients and severe radiological osteoarthritis are variables resulting in the decision by the orthopaedic surgeon to perform a TKA. Symptoms of moderate or severe pain are unequivocal when considering a TKA.

## Electronic supplementary material

Below is the link to the electronic supplementary material.

Supplementary material 1 (DOCX 1590 kb)
