# Supplementary material for: The reason why orthopaedic surgeons perform total knee replacement: results of a randomised study using case vignettes
Source: Knee Surg Sports Traumatol Arthrosc. 2016 Jan 12;24:2697–703. doi: 10.1007/s00167-015-3961-5 (PMC4969334; doi:10.1007/s00167-015-3961-5)
Supplement: Supplementary file 1 — Supplementary material 1 (DOCX 1590 kb) [file 167_2015_3961_MOESM1_ESM.docx]

**Appendix A. Case vignette descriptions.**

**Case 1 Case 2 Case 3**

Medical history

A 54 year * old woman was referred to the outpatient clinic with complaints of progressive knee pain, especially on the left side. No trauma was reported. Start-up pain and morning stiffness are present. She mentioned a VAS pain score of 7. There were no complaints of a locking knee and she is unable to walk more than 30 minutes. She wants to do many activities with her two grandchildren, but she is hindered because of the knee problems.

Conservative treatment
- Painkillers: 3 months NSAID’s with no effect.
- Walking aids: A stick for long distance walks.
- Intra-articular injection: Twice, with a short-term effect.

Physical examination
Minimal varus deformity of the left knee with effusion. Knee-flexion 100 degrees. 5 degree of fixed flexion deformity. Collateral- and cruciate ligaments are stable. Patella no abnormalities.

Standing radiograph knee

**Is a Total Knee Replacement the next step in your treatment?**

* Version B:

**86 years** old.

Medical history
A 68 year old woman is referred to the outpatient clinic and is complaining about pain in both knees, more on the right side. Pain is presented during activities, almost every day. There is no pain at rest or at night while in bed *. Start-up pain and morning stiffness are present. She is incapable of bicycling and has trouble with walking because of the knee problems. This causes great distress in her life.

Conservative treatment
- Painkillers: Minimal effect of NSAID’s.
- Walking aids: Not applicable.
- Intra-articulair injection: Few corticosteroid injections with short-term effect. She does not want the injections anymore.

Physical examination
Minimal varus deformity. Knee-flexion 110 degrees. 5 degree of fixed flexion deformity. Collateral- and cruciate ligaments are stable. Patella no abnormalities.

Standing radiograph knee

**Is a Total Knee Replacement the next step in your treatment?**

* Version B:

**Pain is constantly present including at rest and at night while in bed**.

Medical history
A 67 year old man with left sided knee pain is referred to the outpatient clinic. Pain at rest is present daily, and 2 or 3 times a week he has pain at night. Morning stiffness is present. Maximal walking distance is 1000 metres. It frustrates the patient that bicycling and working in the garden is no longer possible due to the knee problem.

Conservative treatment
Painkillers: Paracetamol 4dd1 gram, if necessary diclofenac 50 mg.
Walking aids: A stick when walking outdoors, for the last 3 months.
Intra-articular injection: He is frightened of injections.

Physical examination
Minimal varus deformity and effusion. Knee-flexion 100 degrees. 10 degree of fixed flexion deformity. Collateral- and cruciate ligaments are stable. Patella-femoral crepitus.

Standing radiograph knee *

**Is a Total Knee Replacement the next step in your treatment?**

* Version B:

** Case vignettes described for version A. The bold letters can be replaced for * (beneath) to obtain version B.*
